# Endorobots for Colonoscopy: Design Challenges and Available Technologies

**DOI:** 10.3389/frobt.2021.705454

**Published:** 2021-07-14

**Authors:** Luigi Manfredi

**Affiliations:** Division of Imaging Science and Technology, School of Medicine, University of Dundee, Dundee, United Kingdom

**Keywords:** medical robotics, colorectal cancer, colonoscopy, endorobotics, design medical devices

## Abstract

Colorectal cancer (CRC) is the second most common cause of cancer death worldwide, after lung cancer (Sung et al., 2021). Early stage detection is key to increase the survival rate. Colonoscopy remains to be the gold standard procedure due to its dual capability to optically inspect the entire colonic mucosa and to perform interventional procedures at the same time. However, this causes pain and discomfort, whereby it requires sedation or anaesthesia of the patient. It is a difficult procedure to perform that can cause damage to the colonic wall in some cases. Development of new technologies aims to overcome the current limitations on colonoscopy by using advancements in endorobotics research. The design of these advanced medical devices is challenging because of the limited space of the lumen, the contorted shape, and the long tract of the large bowel. The force applied to the colonic wall needs to be controlled to avoid collateral effects such as injuries to the colonic mucosa and pain during the procedure. This article discusses the current challenges in the colonoscopy procedure, the available locomotion technologies for endorobots used in colonoscopy at a prototype level and the commercial products available.

## Introduction

1.9 million new cases of colorectal cancer were detected worldwide in 2020 with a mortality of 935 thousand people, 55% male and 45% female (Sung et al., 2021). The World Health Organization (WHO) estimates an average increase rate of 3% per year globally for the next 20 years (Observatory 2021). Early stage detection is key to increase the survival rate, which is close to 100% at Stage 0 and goes down below 5% at Stage IV (Van Erning et al., 2014). Furthermore, early cancer detection permits costs reduction for treatment (Luo et al., 2009).

Screening can be non-invasive (Tepus and Yau 2020) or invasive (Nee et al., 2020). Colonoscopy is the only invasive procedure with the dual capability of optically screen the entire colonic mucosa and perform a polypectomy procedure (Brenner et al., 2014). Interestingly, the removal of polyps is associated with a reduction of 60% of deaths (Fras et al., 2018; Mendivil et al., 2019). Polyps are small clumps of cells that grow on the colonic surface and may eventually become cancer (Gorgun et al., 2016; Ponugoti et al., 2017), thus their removal is an essential procedure able to stop this process.

Colonoscopy is performed by a colonoscope, a long tube with a length of 1.6 m and external diameters ranging from 12 up to 15 mm. It has a camera with light illumination on the tip, a waterjet lens cleaning, air and water insufflation and one or two channels with the dual capability of biopsy and suction to remove residual stool. Colonoscopy procedure causes pain and discomfort to the patient in addition to the bowel preparation required. From the clinician perspective, colonoscopy is a considerable difficult procedure to perform that requires an average training period of four years in the United Kingdom (Siau et al., 2019). Besides, in one over one thousand cases colon perforation can occur during the procedure (Morris et al., 2008).

The colonoscope mechanical design has not changed much in the last 6 decades. It is an expensive device that requires chemical sterilization for decontamination; notwithstanding, it has been reported as a cause of disease transmission (Kovaleva et al., 2013; Kenters et al., 2015; Wang et al., 2018). The average cost of a colonoscopy varies from countries and also between public or private health care center. In the United Kingdom, the NHS has an average tariff cost of £624 (Diagnostic Colonoscopy with Biopsy, 19 years and over), which includes an initial capital investment of about £50,000. Wireless colon capsule (WCC) appeared as an alternative non-invasive screening technique (Ciuti et al., 2020). The first generation, PillCam COLON, was introduced by Given Imaging Ltd. (Yoqneam, Israel) in 2006. It consists of a small capsule with two cameras, one in each distal end, that is swallowed by the patient. An external data system is attached to the body of the patient by a belt where images are recorded. Nevertheless, WCC presents several disadvantages. The procedure is expensive, with an average NHS tariff cost of £611, it requires a more intense colon preparation than traditional colonoscopy (Spada et al., 2012) and the capsule may be difficult to swallow. Moreover, retention can occur (Karagiannis et al., 2009; Rondonotti 2017); the movement of the capsule rely on peristalsis and it cannot be controlled to precisely inspect a particular section of the colonic mucosa. The overall procedure takes more than 8–10 h to screen the large bowel plus additional time to analyze the video (André et al., 2009; Toth et al., 2017; Thygesen et al., 2019). The lack of on-board instruments in this device means that if an abnormal lesion is detected a colonoscopy is still required. Nevertheless, these limitations have not stopped WCC technology to be adopted and being widely used in several medical centers around the world. This is due to the poor acceptance of colonoscopy by patients, the limited access related to the high number of procedures performed per year (Seeff et al., 2004), the long waiting list (Shenbagaraj et al., 2019; Hubers et al., 2020), and the limited workforce to perform colonoscopy.

### Medical Procedure

Analysis of the standard medical procedure is the initial step to design a medical device. It is fundamental to identify the medical needs, the current limitations, and the functionalities that should be incorporated.

Optical examination of the colonic mucosa to identify abnormal lesions is performed by a colonoscopist. Symptomatic or asymptomatic individuals considered at high risk are invited for screening (Christopher et al., 2019). Prior to the procedure, individuals are asked to follow a diet, ingest laxatives and 2 L of solution the night before the procedure to prepare the colon and have a clean environment for the inspection (Sharma et al., 2020). This process is not well accepted (Kelly et al., 2012) and a poor preparation is related to an incomplete colonoscopy (Shah et al., 2007; Brahmania et al., 2012). The procedure, which can last more than 40 min (Jain et al., 2016), starts with the patient lying on a side and the insertion of the colonoscope through the anus, then the rectum and the sigmoid colon. The mobile section of the sigmoid makes this tract very difficult to navigate and it is considered the most painful point (Shah et al., 2002). The patient is asked to rotate from one side to the other to change the resistance as well as the gravity. The procedure consists of a first phase during which the colonoscopist try to reach the cecum, which usually takes less than 20 min (Jain et al., 2016). During this phase, the colonoscope is pushed through the colon applying an external force and torque with the right hand, which provides two degrees of freedom (DOFs) to the instrument. The colonic lumen is expanded and stretched by using insufflation with CO_2_, while water is used to remove any residual stool that may obstruct the inspection. The left hand is used to manipulate two wheels where a cable transmission mechanism allows to control the rotation around two axes of the tip about 10 cm in length with two more DOFs, and a range of motion of approximately up to ±180°. This wide range is required to perform a retroflexion maneuver, whereby the colonoscope looks backward to examinate the distal rectum at the end of the procedure (Rex and Vemulapalli 2013; Kwon and Hahm 2014). This maneuver is very important to increase the adenoma detection rate (EL Shahawy and EL Fayoumy, 2019). The inspection of the colonic mucosa begins after the intubation of the cecum when the colonoscope is withdrawn. This phase requires time to inspect and identify any abnormality in the colonic wall [at least 6 min (Barclay 2017)] and the removal of polyps by using a snare, which is pulled through the instrument channel and collected for biopsy. Tattoo are used to mark this area to enable surgeons to identify the type of colonic resection required (Loeve et al., 2013). The reaction force against the colonic wall allows the instrument to be moved forward. However, this force moves the colon creating loops and stretching the mesenteries, thus causing pain in the procedure (Waye and Thomas-Gibson 2018). Experienced colonoscopists play an important role in reducing pain (Loeve et al., 2013; Lee et al., 2014). Sedation and/or anesthesia are required to reduce pain and discomfort, but they increase the total cost of the procedure and make the patient to stay one or 2 h longer for recovery and risk evaluation, apart from the need to take a day off from work (Wernli et al., 2016).

### Limitations in the Current Procedure

Overcoming the current limitations of the procedure drives research centers and industry to find an alternative solution to colonoscopy (Young et al., 2019). A new technology should satisfy three stakeholders involved in the process, which are (1) patients, (2) clinicians, and (3) operational managers of the endoscopic unit.

*Patients* are reluctant to perform the procedure. As previously mentioned, pain and discomfort are the main reasons to reject it (Holme and Bretthauer 2016). The colon preparation is not well accepted neither easy and difficult to accomplish (Kelly et al., 2012). Poor preparation can lead to missed polyps and/or interval cancer (Kim 2012). Even if conscious-sedation and anesthesia can diminish the pain level, many patients still refuse to undergo colonoscopy, not to mention the higher risk of complications associated to the latter (Cooper et al., 2013; Wernli et al., 2016).

*Clinicians* need a long training to become proficient and the outcome of the procedure relates to their experience and training. The years of experience proportionally correlates with better outcomes (Lee et al., 2014; Siau et al., 2018). This affects the pain and discomfort for the patient as well as the quantity of sedation used during the procedure (Chan et al., 2017). Physical and psychological stress is also a drawback in colonoscopy (Lemke et al., 2019). The posture and fatigue suffered by clinicians during the procedure can cause varying degrees of injuries (Harvin 2014; Austin et al., 2019; Villa et al., 2019).

*Operational managers* strive to implement a more efficient process in the endoscopic unit. The role of an operational manager is to practice a more efficient workflow process to increase the health outcome. According to Porter (Porter 2010), health outcomes can be defined as quality divided by cost. Optimizing this process can be achieved either by increasing quality without increasing cost or reducing cost without compromising quality. Ideally, this can be achieved by simultaneously improve quality while reducing costs. WHO reported that there are no evidence on the efficiency level between private and public healthcare (Hsu 2010). It cannot be generalized which model is the best across countries or within each specific country. The demand on endoscopic services has drastically increased in the past years. This relates to the aging of the population, the increased environmental and behavioral risks, and the changes in the screening policy that may have helped to detect precancerous polyps (Public Health England 2016). The increased number of procedures per year impacts the waiting time for patients, which in turn is affected by a limited workforce (Young et al., 2019). Anesthesia is associated with an increase of risk of complications, colon perforations (Wernli et al., 2016), and costs (Krigel et al., 2019). Cross contamination and additional hidden costs for reprocessing the instruments are additional issues (Larsen et al., 2019). The replacement of the reusable current colonoscope with a disposable device could help to avoid disease transmission (Ciocîrlan 2019).

Due to all these limitations, an alternative low-risk, cost effective, and more efficient solution is needed.

## Endorobots for Colonoscopy

The use of endorobots for medical applications has increased in the last decades and new products have been brought to the market recently. Robotics colonoscopy is one of the procedures that has been widely investigated in research institutes. However, just a few of these results have been effectively translated into the market with FDA approval or CE mark. Limited available products are related to both technical and economic challenges. An innovative technology can definitely improve the patient outcome; however, this can increase the healthcare costs (Leddy et al., 2010; Slakey and Davidson 2019). The high number of colonoscopies performed every year requires a new technology to be cost effective or to bring substantial benefit to the stakeholders. The design of a cost-effective endorobot for colonoscopy needs to solve the high level of engineering challenges that entail fulfilling the necessary requirements.

### Design Requirements

The major advantage of using an endorobot for colonoscopy is the use of a self-propelled force to move the device forward inside the colon and to have a precise control. This is in contrast to the external pushing force of a traditional colonoscope applied by a colonoscopist during the procedure. A self-propelled device can reduce the force applied to the colonic wall, hence reducing the pain and discomfort for the patient. It is expected that if these forces become low enough, at some points, there would be no need of sedation or anesthesia (Korman et al., 2014).

Technical challenges in the design of an endorobot for colonoscopy are: (1) the limited space, (2) the long and tortuous shape, and (3) the slippery surface of the colon mucosa. The diameter of the lumen of the colon varies from 30 mm up to 80 mm when inflated with CO_2_, with a total length of 1.6 m (Alazmani et al., 2016). The current colonoscope has an external diameter that can vary from 12 mm up to 15 mm with dual operational channels. An endorobot is expected to be in the same range without exceeding 20 mm in diameter. To replace the current colonoscope a robotics device should have (1) an effective locomotion solution to inspect all colonic mucosa up to the cecum in about 30 min, (2) a high-definition (HD) camera together with illumination and high-quality video streaming, (3) access to interventional instruments to remove polyps and take biopsy, (4) gas insufflation to expand the lumen to improve the visibility, and (5) waterjet to remove any residual stool from the colonic mucosa. Including all these functionalities in a device represents a challenges and a tether connection to an external console is likely to be required. However, the mechanical stiffness related to the number of wires to be incorporated in the tether, its external diameter, the weight and the friction against the colonic mucosa, produce a drag force that a locomotion system needs to overcome to move the endorobot forward (Ortega et al., 2021). This force has an opposite direction to the locomotion and strongly affects the design of the system and particularly the effectiveness of a locomotion solution.

### Locomotion System

The design of a small and tethered device with a self-propelled locomotion is of paramount importance and requires facing four major challenges: (1) produce enough force to overcome the tether drag; (2) provide a speed to inspect all colonic tract in a time comparable to the current procedure (Jain et al., 2016); (3) reduce the force applied to the colonic wall compared to the colonoscope to reduce pain and discomfort; (4) have a precise control of the device to perform surgical tasks. The light weight of a small device reduces the use of friction-gravity locomotion (F_t_ = *p*·*µ*) due to the low friction coefficient (*µ*) of the mucosa (Ortega et al., 2021) together with a light weight (*p*). This will produce a very limited traction force (Ft).

**FIGURE 1:**
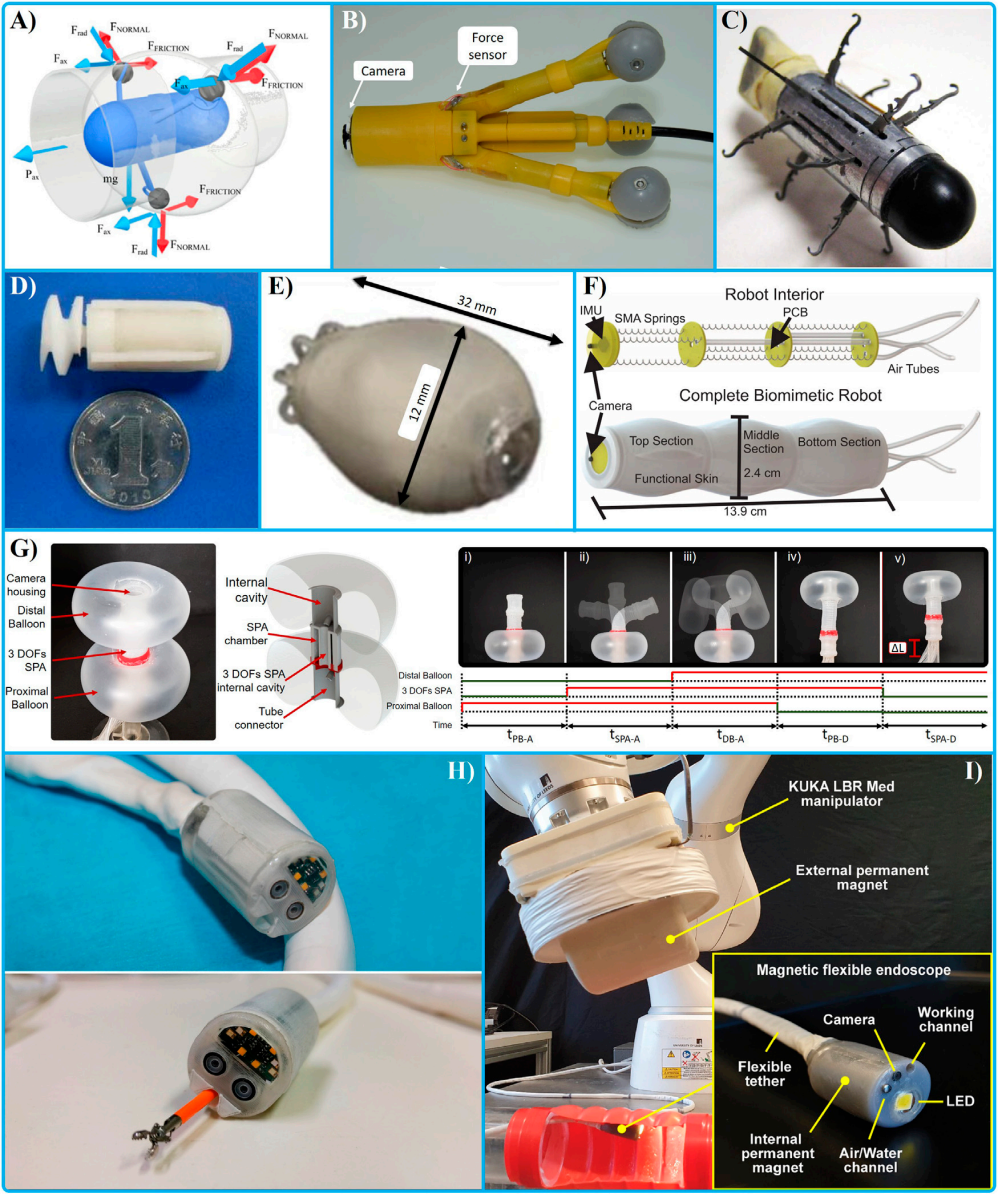
Internal actuators: with wheels **(A, B)** (Karargyris and Koulaouzidis 2015; Norton et al., 2016), **(C)** legs (Valdastri and Webster, 2009), propellers **(D, E)** (Liang et al., 2011; Falco et al., 2014), small inchworm locomotion capsule **(F)** (Alcaide, Pearson, and Rentschler 2017), **(G)** a Soft Pneumatic Inchworm Double-balloon (SPID) (Manfredi et al., 2019); Wireless locomotion: Endoo Project **(H)** (Ciuti et al., 2020) autonomous navigation **(I)** (Martin et al., 2020).

The design of a locomotion system can be classified in: (1) internal or on-board, (2) external, and (3) wireless. Example of available devices are shown in Figure 1.

#### Internal Actuated Locomotion

Several design concepts have been proposed with on-board actuators. Small and miniaturized actuators can be used, such as electric motors, piezo actuators (Kim et al., 2009), electroactive polymers (Mikhaylov et al., 2014), shape memory alloy (SMA) (Manfredi and Cuschieri 2018; Manfredi et al., 2017), or pneumatic (Manfredi et al., 2018; Manfredi and Cuschieri 2018; Manfredi et al., 2019). The small size of the system limits the output force or torque produced by the actuators that can be increased by using a gearbox or a mechanical solution. The low energy efficiency of such actuators requires to take into consideration the dissipation of part of the input energy, which is joule heating. Several designs have been proposed by using DC (direct current) motors combined with wheels **Figure 1A,B** (Karargyris and Koulaouzidis 2015; Norton et al., 2016), (Figure 1C) legs (Valdastri and Webster, 2009), propellers **Figure 1D,E** (Liang et al., 2011; Falco et al., 2014), or continuous circular belts (Formosa et al., 2020). A gear has been used to increase the output torque in a legged device (Valdastri and Webster, 2009). SMAs are known to have low efficiency and low response time (Khan et al., 2016). However, at small scale they can be efficient compared to a small electric motor and present a high mechanical bandwidth while keeping a compact design (Manfredi et al., 2016; Manfred et al., 2017; Manfredi and Cuschieri 2018). Capsules have been proposed by using SMA to implement a small inchworm locomotion (Alcaide et al., 2017) (Figure 1F), with legs and adhesive (Cheung et al., 2005), and suction for an inchworm locomotion (Hosokawa et al., 2009).

Inchworm locomotion has been proposed in several design concepts (Kim et al., 2005). This locomotion consists of a combination between a mechanism to secure anchorage of distal and proximal section of a device together with a mechanical connection to extend the distance between these two sections. The speed (*v*) of an inchworm locomotion relates to the time each step takes to be performed (*Δt*) and the distance covered in each step (*Δl*), *v = Δl/Δt*. The sequence entails five steps: (1) anchorage of the proximal section, (2) extension of the mechanical connection (*Δl*) between the two sections, (3) anchorage of the distal section, (4) contraction and (5) anchorage of the proximal section. Inchworm locomotion has been widely investigated by using different mechanical solutions (Wang et al., 2017), (Hosokawa et al., 2009). Anchoring methods, such as legs (Quirini et al., 2007), vacuum (Cosentino et al., 2009), (Tumino et al., 2017) or balloon (Wang et al., 2013; Chen et al., 2013; Manfredi et al., 2019) have been adopted to increase the contact force and to address the direction of the locomotion. A balloon is an effective solution to increase the contact force while preserving a light weight of the device and low pressure against the contact surface (Wang et al., 2013). Several designs have been proposed to move the device inside a rigid tube (Verma et al., 2018), or a colon with one DOF (Wang et al., 2013) and using a passive bending mechanism relying on force reactions against the surrounding environment. To increase the dexterity of the tip, two toroidal balloons connected by a three DOFs soft pneumatic actuator (SPA) have been proposed to implement a Soft Pneumatic Inchworm Double-balloon (SPID), with an external diameter of 18 mm and total length of 60 mm (Manfredi et al., 2019) (Figure 1G). The dexterity of the SPA can perform a locomotion with an active mechanism to follow the shape of the colon reducing the force applied to the colonic wall. A high number of DOFs in the tip has the dual capability to provide precise control to inspect a particular section of the colon and to control any instrument for surgical tasks. A toroidal balloon can improve stability of a device by fixing his position in the centre of the lumen and avoiding movements during interventional tasks. Patches around the balloon can be used to increase the friction (Chen et al., 2013). A rolling stent has been proposed to keep a continuous motion (Breedveld 2006).

Other locomotion solutions have been proposed, similar to a pipe inspection gauge, by using air (Vucelic et al., 2006) or water (Coleman et al., 2016) to pressurize the entire colon and to propel the device forward like a piston in a cylinder. Waterjet propulsion has been proposed to control the direction of the capsule by using controllable nozzle (Swain et al., 1998; Campisano et al., 2016).

#### External Actuated Locomotion

Locomotion by using external actuators can use heavy and powerful actuators outside the body together with a mechanical transmission such as cables and pulleys to maneuver the device (Eickhoff et al., 2007), or an external shaft (Kim et al., 2014). These solutions have the advantage of increasing the force at the distal part of the device while keeping the design of the internal components small. A snake-like robot with five sections with two DOFs each, controlled by using two DC motors through cables has been investigated. The external diameter is comparable to a colonoscope, 12 mm. However, the total length of 600 mm limits the inspection to the first section of the colon (Hu et al., 2009). For colonoscopy, this design approach needs to take into account the length of the transmission and the tortuous shape of the colon. This configuration can cause friction in the cable transmission limiting the output force (Agrawal, Peine, and Yao 2010). Cable actuation also increases the stiffness of the tether. Hydraulic actuation systems can be also used, however, high pressure inside the transmission tubes due to the small cross section of the device (Cuntz and Comella 2015) can pose safety concerns.

#### Wireless locomotion

Wireless locomotion can be achieved by means of a magnetic field. Magnetic actuation for endoscopic robots is an intriguing design approach that can be implemented with a small permanent magnet inside the device and a magnetic field produced by an external apparatus. Several works have produced small devices controlled by permanent external magnets together located in a robotic arm to control the orientation and the magnetic field (Yim and Metin, 2012), (Ciuti et al., 2020) (Figure 1H). To increase the locomotion force, neodymium magnets are often used. An external permanent magnet has a limited volume compared to a coil and can produce force at higher distance. However, the use of a coil can improve the controllability of the target device (Edelmann et al., 2018). The control can be improved by using a location solution to implement a closed-loop controller on the locomotion (Taddese et al., 2018). To reduce the contact force and friction of the capsule against the colonic wall, a dynamic control to achieve a magnetic levitation has been developed (Pittiglio et al., 2019). The use of an external robotic arm that moves a permanent magnet requires additional space in the control console. This also increases the cost of the platform since the arm needs to fulfill all the medical regulations (Leenes et al., 2017). Endoo (Endoscopic versatile robotic guidance, diagnosis and therapy of magnetic-driven soft-tethered endoluminal robots), a European Project funded by an EU H2020 grant (2015–19), led by the Scuola Superiore di Studi Universitari e di Perfezionamento Sant’Anna (Endoo EU H2020 Project, 2015), developed a magnetic actuated platform to perform colonoscopy with high-quality camera, biopsy instruments and a soft-tether (Verra et al., 2020). The NaviCam capsule endoscope include a magnetic control system and a wireless capsule (28 × 12 mm). This is a commercially available device proposed by Ankon Technologies Co., Ltd., Wuhan in China (Liao et al., 2016). The capsule has an angle of view of 140°, and a view distance up to 60 mm. This device has no on-board instrumentation. To improve the locomotion force and dexterity, a wireless capsule has integrated the magnetic field with an on-board DC motor connected to a screw mechanism (Wang et al., 2010). Algorithms for an autonomous lumen detection for an autonomous navigation have been developed and validated (Martin et al., 2020) (Figure 1I).

## Available Products

**FIGURE 2:**
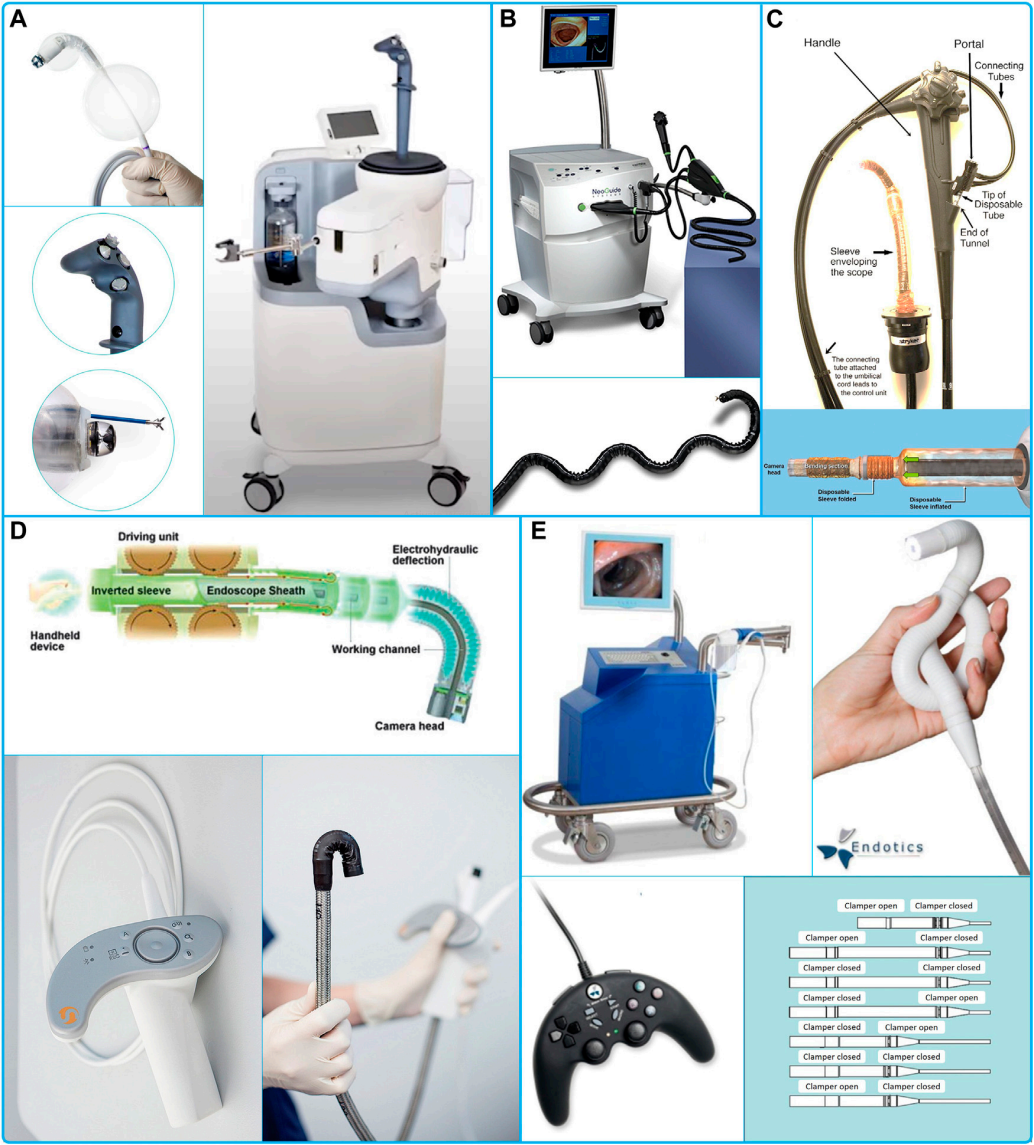
**(A)** Aer-o-Scope GI-View (Aer-O-Scope Colonoscope, 2021), **(B)** Neoguide System (Karimyan et al., 2009), **(C)** ColonoSight (Sightline Technologies Ltd. Haifa, Israel) (Shike et al., 2008), **(D)** Invendo Medical GmbH (Kurniawan and Keuchel 2017), **(E)** Endotics® System from Era Endoscopy (Cosentino et al., 2011).

Just few devices that have incorporated biopsy channel have achieved an advanced development stage to be granted CE mark or FDA 510 (k) certificates (Figure 2).

Aer-o-Scope GI-View (GI View Ltd. Ramat Gan, Israel) (Vucelic et al., 2006), (Aer-O-Scope Colonoscope, 2021) (Figure 2A) has been granted CE mark and FDA 510 (k) in 2016, but the product is not yet available. The device includes an external joystick controller with automatic pressure management, a 360° camera for an omni-directional visualization to improve the visibility and polyp detection rate. It is single use with two working channels. The device entails a balloon that after insertion seals the colon. Then CO_2_ pressurizes the colon and propels the device forward, to seal the distal section with one more balloon. The external workstation limits the internal pressure to 60 mbar (Gluck et al., 2016). Two studies, one with a proof-of-concept of the device (Vucelic et al., 2006), and the most recent with a more advanced device with no working channels (Gluck et al., 2016), successfully completed cecum intubation with no need of sedation. The GI-View website indicates that similar sedation to that performed for a conventional colonoscopy is required (Aer-O-Scope Sedation, 2021).

Moreover, cable actuation has been used in the NeoGuide Endoscopy System (NeoGuide Endoscopy System Inc. Los Gatos, CA United States), a device granted the 510(k) clearance in 2006 (Eickhoff et al., 2007) (Figure 2B).

ColonoSight (Sightline Technologies Ltd. Haifa, Israel) (Figure 2C) system received FDA approval in 2004 (Model 510B) and then acquired by Stryker GI Ltd. (Haifa, Israel) in 2006. The device is disposable, and the locomotion is provided by the air inflated inside a sleeve that covers an inner tube (Shike et al., 2008). The tip has a bendable section, a camera, biopsy channel, suction, insufflation and irrigation channels.

Invendo Medical GmbH is a German based company acquired by Ambu A/S in 2017 (Invendo Acquisition, 2017) (Figure 2D). They have proposed several iterations of their robotic colonoscope. Starting with the SC40 (Rösch et al., 2008), then SC200 with CE mark and FDA 510 (k) clearance in 2017, and finally an upgraded version, the E210, FDA 510(k) in 2018. The propulsion is provided by a double layer of an inverted sleeve. The inner sleeve is actuated from outside by eight wheels that drives the device forward through their rotation, causing the device to “grow” and avoiding relative movement of the tip and reducing forces applied to the colonic wall. The device has a working channel of 3.1 mm, external diameter of 18 mm, total length of 2,100 mm, field of view of 114° (Groth et al., 2011) and an electrohydraulic actuation for the tip.

For its part, Endotics® System from Era Endoscopy, Peccioli, Italy, provides a single use colonoscope with a biopsy channel and an inchworm locomotion (Figure 2E). The anchorage is provided by a suction mechanism followed by a clamp to the colonic wall. The locomotion has an auto-locomotion functionality. This device received the CE mark in 2017 and FDA 510(k) approval in 2020. Endotics is, so far, the only available robotic device in use.

## Discussion

Introducing a new technology in publich health has always been challenging. In the United Kingdom, value for money of a new medical device is assessed through a process of Health Technology Assesment (HTA). The National Institute for Health and Clinical Excellence (NICE) and the National Coordinating Center for Health Technology Assessment (NCCHTA) are the key national HTA organizations. NICE plays the most important role to introduce a new technology in the health care system. This requires to verify the medical device from safety to efficacy through to patient system and economic benefit.

Robotics technology can improve the current procedure by proposing an alternative low-risk and, in some cases, cost effective solution. Several companies in the last two decades have embraced the challenge and brought innovative solutions to the market. Clinical trials have proved important advantages of these devices vs. current technologies, including lower pain, no need of sedation, and the possibility to be disposable. Certified agencies have confirmed satisfaction standards for health, safety, and environmental protection. However, limited numbers of procedures are currently performed by these devices. These limitations can be related to some “hidden” issues in the technology that has repressed their application in clinical practice. Other factors can be related to the marketing and commercial strategies, which were not able to place the product and convince the stakeholders to adopt it. Colonoscopy is an area that needs innovation, and the update of the current and dated colonoscope requires a big effort that involves substantial changes in an increasing complex healthcare environment.
